# Epispadias masculin isolé

**DOI:** 10.11604/pamj.2017.28.202.14220

**Published:** 2017-11-06

**Authors:** Yddoussalah Othmane, Alae Touzani

**Affiliations:** 1 Service d’Urologie B, CHU Ibn Sina, Faculté de Médecine et de Pharmacie de Rabat, Université Mohamed V, Maroc

**Keywords:** Epispadias, verge, prise en charge, Epispadias, penis, management

## Abstract

Epispadias is a rare urogenital malformation characterized by more or less complete aplasia of the upper part of the urethra. It is commonly associated with bladder exstrophy. Isolated epispadias occurs in 10% of cases. Continent and incontinent epispadias may be distinguished. We report the case of a 29-year old patient presenting with dysuria associated with the impossibility of having sexual intercourses. Physical examination of the external genitalia showed slit-like abnormal urethral opening on the distal half of the dorsal side of the penis. Patient’s penis didn’t have dorsal curvature (chordee), the corpus cavernosum was palpated and slightly lateralized. The abdominal wall was without abnormalities. The remainder of the clinical examination was normal. Given this anomaly, the diagnosis of balano-pubic continent epispadias was retained. Ultrasound of the urinary tract was normal and pelvis X-ray without preparation showed interpubic diastasis. The patient underwent single-stage surgery using Cantwell-Young technique. Immediate postoperative outcome was without abnormalities; the urethral probe was removed 21 days later, after wound healing. Functional and aesthetics outcome evaluated at three and six months was satisfactory without penis shortness.

## Image en médecine

L’épispadias est une malformation urogénitale rare se caractérisant par une aplasie plus ou moins complète de la face supérieure de l’urètre. Fréquemment associé à une exstrophie vésicale, il est isolé dans 10% des cas. On distingue donc des épispadias continents et incontinents. Nous rapportons le cas d’un patient âgé de 29 ans, qui consulte pour prise en charge d’une dysurie et de l’impossibilité d’avoir des rapports sexuels. À l’examen, des organes génitaux externes notaient une ouverture anormale de l’urètre sous forme de fente sur la moitié distale de la face dorsale de la verge. La verge ne présentait pas de courbure dorsale (chordée), les corps caverneux étaient palpés, légèrement latéralisés. La paroi abdominale était sans anomalie. Le reste de l’examen était normal. Devant cette anomalie, le diagnostic d’épispadias continent balano-pubien a été retenu. Une échographie de l’arbre urinaire réalisée est revenue normale et une radiographie sans préparation du bassin montrait un diastasis inter pubien. Le patient a été opéré en un temps selon la technique de Cantwell-Young, l'évolution postopératoire immédiate était sans anomalie; la sonde urétrale a été enlevée 21 jours plutard après cicatrisation. Le résultat fonctionnel et esthétique évalué à trois et à six mois ont été jugés satisfaisants sans brièveté de la verge.

**Figure 1:**
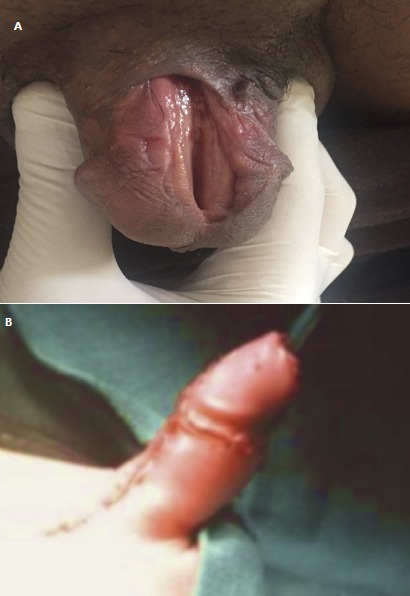
A) épispadias balanopénien continent chez sujet de 29 ans avec exposition de la plaque urétrale au niveau du gland: aspect pré opératoire; B) aspect post-opératoire immédiate d’un épispadias chez un homme

